# Intraoperative adaptive eye model based on instrument-integrated OCT for robot-assisted vitreoretinal surgery

**DOI:** 10.1007/s11548-025-03325-0

**Published:** 2025-02-08

**Authors:** Marius Briel, Ludwig Haide, Maximilian Hess, Jan Schimmelpfennig, Philipp Matten, Rebekka Peter, Matthias Hillenbrand, Eleonora Tagliabue, Franziska Mathis-Ullrich

**Affiliations:** 1 Carl Zeiss AG, Oberkochen, Germany; 2 Laboratory for Surgical Planning and Robotic Cognition (SPARC), Friedrich-Alexander-University, Erlangen, Germany

**Keywords:** Ophthalmic surgery, Eye models, Optical coherence tomography, Intraoperative assistance

## Abstract

**Purpose:**

Pars plana vitrectomy (PPV) is the most common surgical procedure performed by retinal specialists, highlighting the need for model-based assistance and automation in surgical treatment. An intraoperative retinal model provides precise anatomical information relative to the surgical instrument, enhancing surgical precision and safety.

**Methods:**

This work focuses on the intraoperative parametrization of retinal shape using 1D instrument-integrated optical coherence tomography distance measurements combined with a surgical robot. Our approach accommodates variability in eye geometries by transitioning from an initial spherical model to an ellipsoidal representation, improving accuracy as more data is collected through sensor motion.

**Results:**

We demonstrate that ellipsoid fitting outperforms sphere fitting for regular eye shapes, achieving a mean absolute error of less than 40 $\upmu \hbox {m}$ in simulation and below 200 $\upmu \hbox {m}$ on 3D printed models and ex vivo porcine eyes. The model reliably transitions from a spherical to an ellipsoidal representation across all six tested eye shapes when specific criteria are satisfied.

**Conclusion:**

The adaptive eye model developed in this work meets the accuracy requirements for clinical application in PPV within the central retina. Additionally, the global model effectively extrapolates beyond the scanned area to encompass the retinal periphery.This capability enhances PPV procedures, particularly through virtual boundary assistance and improved surgical navigation, ultimately contributing to safer surgical outcomes.

## Introduction

Pars plana vitrectomy (PPV) is a routinely performed procedure in eye surgery that consists of partial or complete removal of the vitreous humour, i.e. the gelatinous tissue that fills the space between the lens and the retina. PPV is typically conducted as a preliminary procedure in various retinal surgeries to facilitate access to the retina. It includes core vitrectomy, posterior hyaloid detachment, and peripheral vitrectomy. The risk of retinal damage during core vitrectomy is minimal due to the central positioning of the instruments. In contrast, peripheral vitrectomy presents a higher risk, as it involves high-speed cutting in proximity to the retina. Robot-assisted PPV has the potential to facilitate this delicate step while reducing the surgeon’s workload, by maintaining a safe distance from critical structures or providing an optimal instrument path.

One of the challenges of patient-specific assistance is that eyes vary in size by millimeters. In addition, some eyes are significantly deviating from their natural shape due to conditions such as retinal detachment or staphyloma. Therefore, the implementation of assistance functions must be based on patient-specific data. Eye geometry data could be acquired prior to surgery from magnetic resonance imaging (MRI) [1, 2], computed tomography (CT) [3], or optical coherence tomography (OCT) [4]. However, the use of pre-operative scans would require accurate registration to the surgical instrument coordinate frame, which is challenged by the scarcity of geometric retinal features, potential inter-scan tissue deformations, and geometric distortions present in the pre-operative scans [5], thus making intraoperative sensing desirable.

Intraoperative retinal imaging includes surgical microscopy [6], ophthalmic endoscopy [7], and OCT [8]. Zhang et al. [9] have leveraged microscope images to safely navigate a surgical instrument to a desired location on the retina with optimal control, but did not evaluate the local ellipsoid fitting system component. Moreover, stereo reconstruction from microscope images is challenging due to distortions caused by the anterior segment of the examined eye, strong illumination changes, and hemorrhages [6]. A robotic ophthalmic endoscope can be used to build a retinal representation by combining the kinematics of the robot with the distance information through non-stereoscopic cues from the endoscope [10]. The main limitation of their work - besides the relatively large reconstruction error of 0.5  $\hbox {mm}$ - is that most eyes cannot be accurately modeled as spheres [1–3]. OCT is widely used in both ophthalmic diagnostics and surgery and provides excellent image quality. Intraoperative 4D OCT can be used directly for tool-to-layer distance estimation [11] in the central retina if distortion effects are neglected.

Another promising solution for intraoperative sensing is fiber-based intra-ocular OCT, commonly called instrument-integrated OCT. An optical fiber is attached to or inserted into a surgical instrument that is connected to a surgical robotic arm. It continuously acquires axial scans as the robot moves the instrument inside the eye. iiOCT-based distance sensors for robotic vitreoretinal surgery were first clinically validated by Cereda et al. [12]. iiOCT was used by Balicki et al. to define distance-based virtual boundaries that force the surgeon to maintain a certain distance from the retina  [13]. In addition, iiOCT has also shown promise in preventing retinal tears by deactivating the vitrectome cutter just before cutting the retina [14]. To realize real-time safety features for PPV, it is essential to perform online OCT signal processing and segmentation to obtain intraocular distances.

**Fig. 1:**
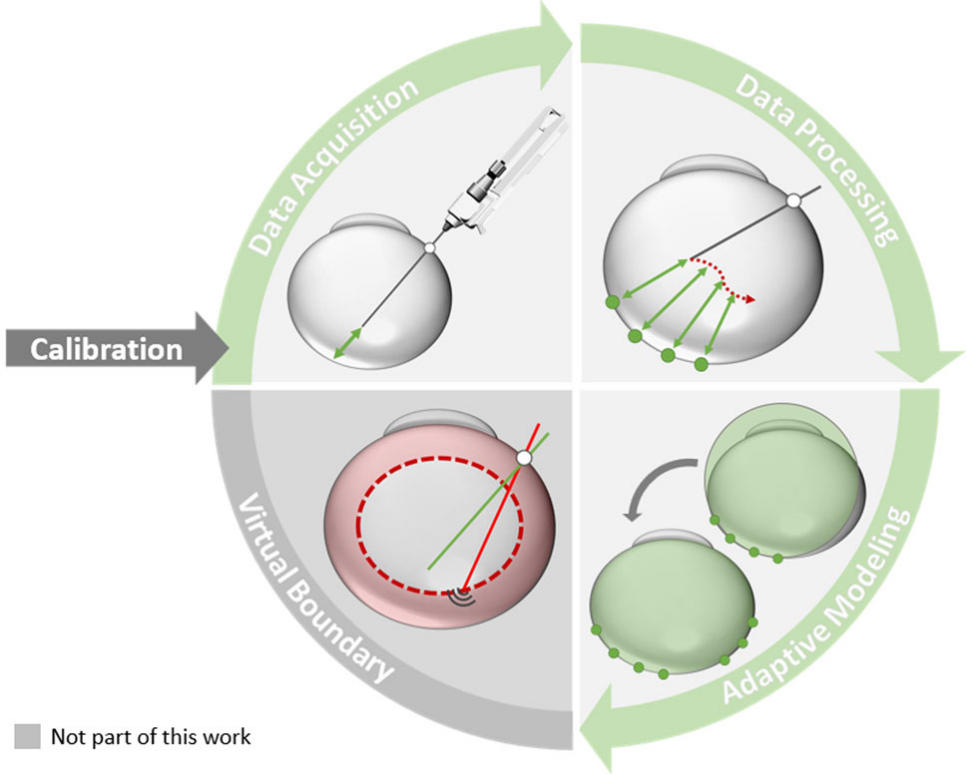
Method overview: Instrument-integrated optical coherence tomography (iiOCT) pose calibration, acquisition of iiOCT data and robot kinematics, segmentation of iiOCT images and synchronization of the two sensor streams to reconstruct the retina, intraoperative adaptive modeling of the retinal boundary with a transition from sphere to ellipsoid when appropriate, and the future use of such a model for a virtual boundary (not implemented)

iiOCT scanning is unlikely to be sufficient for intraoperative assistance, as it only provides information in the axial direction and not in the lateral direction of the aspiration port. However, a model can provide information about unscanned areas and anticipate lateral distances. It also has the potential to supplement measurement when distance extraction from measurement scans fails, or when excessive sensor noise is present. The sole existing prior research on iiOCT-based eye models is that of Cornelissen et al. [15]. However, only sphere fitting is performed, and no validation is conducted on more realistic geometries or ex vivo eyes. Additionally, they lack sensor pose calibration.

In this work, we take a first step towards model-based assistance during vitrectomy by developing a global eye model that dynamically adapts intraoperatively to the geometry of the individual patient’s retina based on iiOCT data. In particular, our contributions are:

1. We propose a patient-specific model that approximates the shape of the retina by transitioning from sphere to ellipsoid and maximizing model reliability based on the available iiOCT data.
2. We show that the model meets accuracy and time requirements that make it applicable within the standard clinical workflow.
3. We validate the method in a real-world robotic setup using 3D printed and ex vivo porcine eyes.

## Method

We create a global model of the eye based on the retinal boundary points extracted intraoperatively from iiOCT and the robot kinematics (see Fig. 1).

Axial distances to the retinal surface are determined through the analysis of intensity profiles in iiOCT A-scans and template matching [16]. Assuming a similar intensity-value distribution between A-scans, distances are calculated by identifying the index corresponding to the maximum cross-correlation of each A-scan with a reference A-scan. The initial distance is derived from a peak search that measures the disparity from the fiber tip of the OCT sample arm to the most prominent interface.

The robot kinematics provides the pose of the flange, the last joint of the robot. The iiOCT pose at the flange is determined through calibration. To reconstruct retinal points from the measured instrument-to-retina distances, the axial distances are added to the sensor position along its direction.

Our methodology is designed to accommodate the variability in eye shapes by initially applying a robust sphere fitting technique. As more data is collected through sensor motion, the adaptive model transitions to an ellipsoidal shape.

### Sphere fitting

A sphere is parameterized by its center point $(x_0, y_0, z_0)$ and its radius *r*. The online phere fitting (SF) minimization problem can be derived from the sphere equation as

1 $$\begin{aligned} \begin{pmatrix} x_i \\ y_i \\ z_i \\ 1 \end{pmatrix} \cdot \begin{pmatrix} 2x_0\\ 2y_0 \\ 2z_0 \\ r^2 - x_0^2 - y_0^2 - z_0^2 \end{pmatrix} - \left( x_i^2 + y_i^2 + z_i^2 \right) , \end{aligned}$$

to obtain linear dependencies between the given measurement point $(x_i,y_i,z_i)$, and the sphere parameters $( x_0, y_0, z_0, r)$ which are to be optimized. For online fitting, the measurements of the eye are acquired progressively once the iiOCT is inserted into the eye. Online algorithms can be used to continuously update and improve the model. To solve this online optimization problem, we used a Kalman filter, following the same approach as Cornelissen et al. [15].

### Ellipsoid fitting

Every ellipsoid $\mathcal {E}$ satisfies

2 $$\begin{aligned} \mathcal {E} = \{ R D \vec {\eta } + \vec {\omega }: \vec {\eta } \in S^2 \} \end{aligned}$$

for some positive definite diagonal matrix *D* with diagonal entries $d_i > 0$, an orthogonal rotation matrix *R*, and a center vector $\vec {\omega } = \left( x_0, y_0, z_0 \right)$. *D* determines the lengths of the ellipsoid’s principal axes and *R* its orientation. Direct Direct ellipsoid fitting (EF) requires solving a nonlinear optimization problem that often stops at a local minimum. Instead, we minimize the general quadratic equation

3 $$\begin{aligned}&ax^2 + by^2 + cz^2 + 2fyz + 2gxz + 2hxy \nonumber \\&\quad + 2px + 2qy + 2rz + d , \end{aligned}$$

using the method of least squares, to avoid nonlinear optimization. However, an additional nonlinear constraint

4 $$\begin{aligned} 4(ab + bc + ac - f^2 - g^2 - h^2) - (a+b+c)^2 > 0 \end{aligned}$$

on the coefficients (*a*, *b*, *c*, *f*, *g*, *h*, *p*, *q*, *r*, *d*) is required to specifically fit ellipsoids [9, 17].

We subsequently recover the parameters *D*, *R*, and $\vec {\omega }$ that geometrically describe an ellipsoid. The matrices *R* and *D* are obtained from the eigenvalues $\lambda _i$ and right eigenvectors of the parameter matrix

5 $$\begin{aligned} A = \begin{pmatrix} a &  h &  g \\ h &  b &  f \\ g &  f &  c \end{pmatrix} . \end{aligned}$$

We set $d_i = 1 \backslash \sqrt{\vert \lambda _i \vert }$ and the corresponding eigenvectors as the *i*-th columns of *R*. The center point is obtained as

6 $$\begin{aligned} \vec {\omega } = - A^{-1} \begin{pmatrix} p \\ q \\ r \end{pmatrix} . \end{aligned}$$

By scaling the unit sphere $S^{2} = \{\vec {\eta } \in \mathbb {R}^3: \Vert \vec {\eta } \Vert = 1\}$ with *D*, rotating with *R*, and translating with $\vec {\omega }$, one obtains the fitted ellipsoid [18]. The fitted ellipsoid point cloud can be generated using spherical coordinates with any desired discretization.

### Model transition

SF is a linear optimization problem without constraints, which makes the optimization robust even when the input points are concentrated near a small area of the sphere’s surface. On the other hand, an ellipsoid leads to a difficult to solve optimization problem, especially when the data is scarce, noisy, and non-uniform. In light of the aforementioned considerations, we propose a transition from a spherical model to a more realistic ellipsoid model as soon as the amount and quality of data available allow for such a transition.

We consider three different empirical model transition criteria, and propose to transition from sphere to ellipsoid once all of them are fulfilled. *Scan range* is defined as the greatest distance between two measured retinal points. Transition is allowed once the scan range is above 20 $\hbox {mm}$. *Model confidence* is defined as the SF and the EF residuals. A transition can occur if the model confidence is greater for the ellipsoid than for the sphere. *Temporal ellipsoid variation* is defined as the relative change in the ellipsoid axes between two consecutive model updates. The criterion prompts the model to transition when the maximum variation of all semi-axes is less than $0.03\%$ for more than 15 consecutive iterations. The values were determined experimentally to meet our requirements.

## Experimental validation

### Experimental setup

**Fig. 2:**
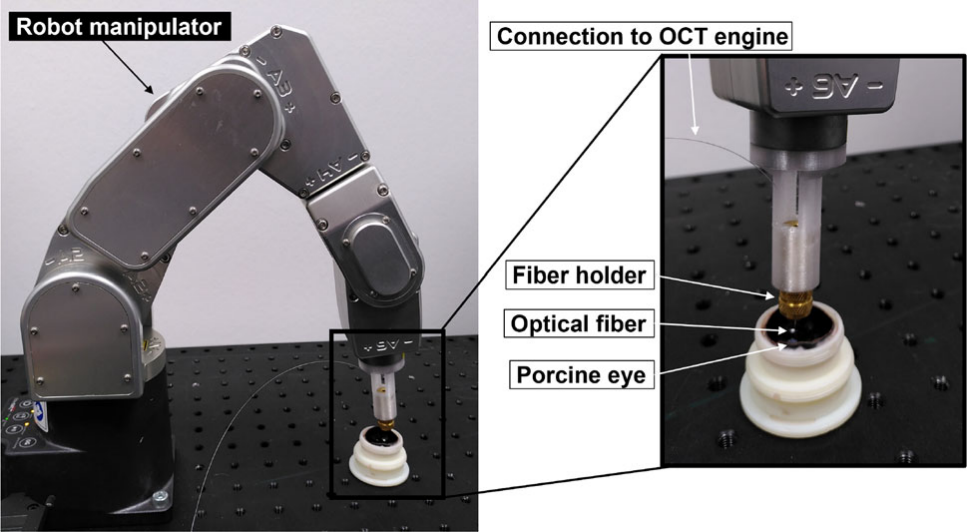
The experimental setup comprises a Meca500 industrial robot and an optical fiber coupled with a swept-source OCT engine

This section presents a description of the principal components of the experimental setup, as illustrated in Fig. 2.

#### OCT-based distance sensing

The IOL Master 700 biometry device (ZEISS, Germany), which is equipped with a 2 $\hbox {kHz}$ high-speed swept-source laser engine with a center wavelength of 1060 nm, is used as OCT engine. It provides an imaging depth of approximately 60 $\hbox {mm}$ in air and an axial resolution of 30 to 40 $\upmu \hbox {m}$.

We utilize a custom sample arm fiber (HI-1060, LaseOptics, USA), equipped with a 300 $\upmu \hbox {m}$ diameter focusing ball lens with 2 $\hbox {mm}$ working distance at the tip. The end of the fiber is stripped and incorporated into a protective stainless steel ferrule with a 760 $\upmu \hbox {m}$ outer diameter and 15 $\hbox {mm}$ length.

#### Robotic motion control

The Meca500 (Mecademic, Canada) is a 6 degrees of freedom (DOF) robotic arm with motion repeatability of 5 $\upmu \hbox {m}$. Trajectories are robotically executed for repeatability and commands are sent to the robot via robot operating system (ROS).

The iiOCT tip is moved on a parametrized Archimedean spiral. The curve is comparable to the movements observed during core vitrectomy, during which the model is constructed. Consequently, we anticipate that the model construction will exhibit similar behavior when utilizing data derived from actual surgeon movements instead of the spiral scan. The spiral

7 $$\begin{aligned} f{:}\theta \mapsto \left( b\, \frac{\theta }{2\pi }\cos \theta ,b\, \frac{\theta }{2\pi }\sin \theta , 0\right) \end{aligned}$$

commences at the center of the eye and is aligned with the coronal axis. Parameter $b \in \mathbb {R}$ controls the distance between loops and is set to $b=0.5$ mm. For 18 revolutions, we set $\theta \in [0,36\pi ]$. Knowing the length of the spiral, we choose $\theta$ in such a way that the points on the spiral are equispaced with a distance of 0.1 $\hbox {mm}$ between consecutive points.

#### Distance sensor calibration

The optical fiber is attached to a 3D printed mount using a fiber holder to interface with the Meca500 robotic arm. Precise knowledge of the iiOCT pose relative to the robot end effector is essential for reconstructing retinal points from iiOCT measurements. To accurately estimate such a transformation, we adapt the calibration procedure proposed by Sifferman et al. [19]. To comply with the kinematic constraints in vitreoretinal surgery, we impose a remote center of motion (RCM) at the trocar, i.e. the point on the sclera of the eye where the instrument is inserted into the intraocular space.

**Fig. 3:**
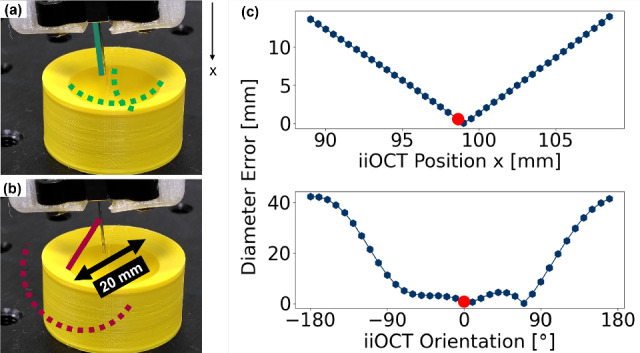
**a** If the sensor pose (green solid line) is calibrated, the true size and position of the estimated sphere can be obtained. **b** An incorrect sensor pose relative to the robot end effector results in an incorrect size and position (red dashed arc). **c** The calibrated sensor pose (red dot) yields superior SF results in terms of absolute diameter error when compared to other sensor poses (blue dots)

We measure the distance to a static OCT calibration plate from 250 different robot poses. We then determine the iiOCT pose with a sequential least squares programming (SLSQP) optimization approach using the known pose of the end effector and the condition that all measured points must lie on one plane. Calculations were performed on an Intel Core i5 processor. The calibration yielded an average residual error from the plane of less than 30.03 $\upmu \hbox {m}$. We also perform a sensitivity analysis to understand how critical the extrinsic sensor calibration is to the sphere fitting, as shown in Fig. 3. The sensor pose is systematically perturbed in the vertical direction and rotated in the image plane.

**Table 1:**
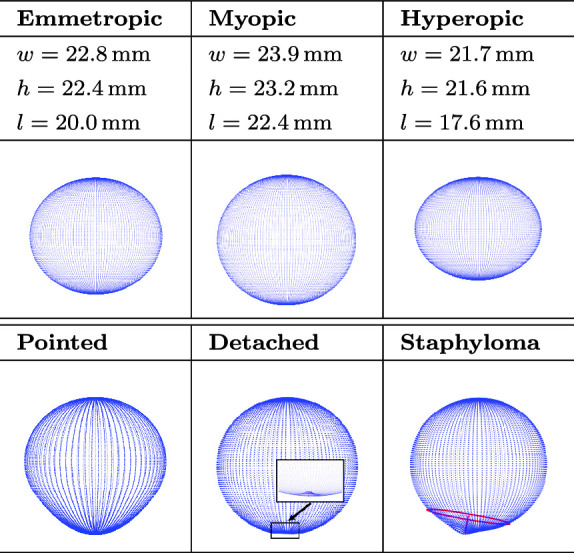
The realistic test eye shapes considered for evaluation of our approach

#### Test eyes

Six different eye shapes are constructed as point clouds based on parameters found in the literature (Table 1) and are used in both simulation and real-world testing of the fitting algorithms. The selected eye shapes are intended to represent both common eye geometries, including myopia and hyperopia, as well as pathological conditions such as staphyloma and retinal detachment. A staphyloma is a circumscribed protrusion at the back of the eye. A retinal detachment can occur when the retina breaks and the vitreous leaks into the subretinal space.

The average emmetropic eye is oblate [1]. As myopia increases, eyes become larger in width (0.14 $\hbox {mm}/\textrm{diopter}$) and height (0.10 $\hbox {mm}/\textrm{diopter}$), but even more so in length (0.30$\,\hbox {mm}/\textrm{diopter}$) [2]. Almost half of highly myopic eyes have a pointed shape in the posterior part [20]. Hyperopia, like myopia, is axial in nature [21]. Here, we assume that the relationship between height, width, and length, as previously mentioned, also applies to hyperopia. We assume a visual acuity of −12 diopters for all pathologies because highly myopic patients are considerably more likely to develop retinal detachment, staphyloma, or pointedness.

The lower halves of all six eye shapes in Table 1 are converted to.stl files and 3D printed with Extrudr PLA NX2 filament on an i3 MK3S+ printer (Prusa, Czech Republic). The layer height is 0.05 $\hbox {mm}$. The ex vivo porcine eyes are cut in half (*open sky*) and placed in a spherical eye holder with an inner diameter of 13 $\hbox {mm}$. The vitreous, which is firmly attached to the optic nerve head, is removed with a scraper (*dry*).

### Experiments

**Fig. 4:**
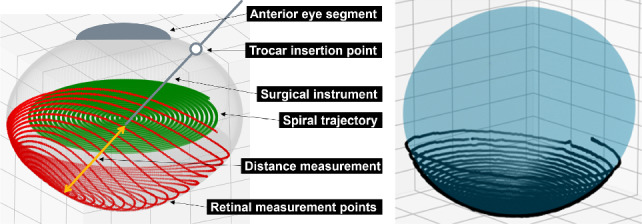
Simulation environment that simulates iiOCT measurements to reconstruct retinal points based on different robot trajectories (left) and real-world measurement points of a hyperopic eye phantom, along with the corresponding ellipsoid fit (right)

As a first evaluation, we test the performance of our modeling approach and transition strategies using the defined eye shapes from Table 1 in a simulation environment (Fig. 4). This strategy enables a quantitative evaluation of the modeling component, as accurate reference geometry is available and independent of inaccuracies arising from sensor, calibration, 3D printing, and registration.

The instrument tip is modeled as a point in space and the instrument is modeled as a straight line connecting the instrument tip and the trocar. The simulated distance measurement calculates the intersection point between the iiOCT beam and the reference retina. To simulate real conditions, we perturbed the simulated distance measurements with a normal distribution with a standard deviation of 20 $\upmu \hbox {m}$, which is in the range of the axial resolution of our sensor.

For data acquisition in the real world, the six printed eye shapes and three ex vivo porcine eyes are utilized. The RCM is positioned along the visual axis for both the pig and the printed eyes, due to the limited height of the shapes to be scanned. To synchronize the OCT stream with the robot encoder data, a unique robot trajectory is executed in the form of an amplitude modulated sinusoidal signal. The synchronization results in a time deviation between the time series of less than 2.6 $\hbox {ms}$.

### Evaluation metrics

The mean absolute error (MAE) serves to quantify the discrepancy between the fitted sphere or ellipsoid and its corresponding ground truth, thereby enabling an assessment of the overall accuracy of the robotic measurement system and the modeling. The measurement mean absolute error (MMAE) represents the deviation of the measurement points from the ground truth shapes, thereby enabling the assessment of the inaccuracies of the measurement setup. It can be regarded as a benchmark that the MAE cannot surpass, irrespective of the quality of the fitting. The fitting mean absolute error (FMAE) represents the deviation of the data points from the fitted shapes, thereby enabling the assessment of the accuracy of the SF and the EF within the scanned region. In contrast, the MAE is evaluated on a global scale.

To calculate the MAE, the MMAE and the FMAE, the model and the ground truth are sampled with $500 \times 500$ points. In order to account for the fact that the retina does not represent a full ellipsoid due to the presence of the anterior eye segment, the anterior five millimeters in length direction are cropped.

For the evaluation of fitting on the printed shapes, the ground truth geometry of the eye is available, but the absolute position is unknown. Consequently, the fitted point cloud is aligned to the reference using a registration based on iterative closest point (ICP). The implementation from Open3D was used, and the maximum correspondence distance was varied between 0.1 and 1.0. In the absence of a ground truth geometry, we calculated only the FMAE for the porcine eyes.

## Results

The results pertaining to the measurement errors, sphere and ellipsoid fitting errors, and overall errors, as well as their standard deviation (SD), are presented in Table 2.

**Table 2:** Errors (MMAE, FMAE, and MAE) and SD of sphere fitting (SF) and ellipsoid fitting (EF) in simulation (top) and on 3D printed shapes (bottom)

	MMAE [mm]	SF		EF	
		FMAE [mm]	MAE [mm]	FMAE [mm]	MAE [mm]
Emmetropic	0.041 (0.016)	0.079 (0.040)	0.244 (0.277)	**0**.**038** (0.015)	**0**.**035** (0.016)
Myopic	0.043 (0.016)	0.078 (0.039)	0.147 (0.123)	**0**.**040** (0.016)	**0**.**038** (0.016)
Hyperopic	0.039 (0.015)	0.099 (0.052)	0.309 (0.355)	**0**.**036** (0.014)	**0**.**033** (0.015)
Pointed	0.068 (0.045)	**0**.**178** (0.138)	**0**.**401** (0.285)	0.569 (0.114)	0.744 (0.293)
Detached	0.043 (0.016)	0.090 (0.049)	0.147 (0.127)	**0**.**043** (0.022)	**0**.**061** (0.070)
Staphyloma	0.043 (0.016)	0.098 (0.066)	**0**.**168** (0.161)	**0**.**069** (0.057)	0.232 (0.287)
Emmetropic	0.100 (0.070)	**0**.**090** (0.064)	0.274 (0.359)	0.178 (0.049)	**0**.**179** (0.105)
Myopic	0.078 (0.047)	**0**.**100** (0.068)	0.208 (0.244)	0.147 (0.053)	**0**.**165** (0.128)
Hyperopic	0.096 (0.071)	**0**.**090** (0.059)	0.371 (0.514)	0.208 (0.046)	**0**.**197** (0.113)
Pointed	0.265 (0.162)	0.262 (0.175)	**0**.**499** (0.793)	**0**.**162** (0.086)	0.810 (0.665)
Detached	0.109 (0.077)	**0**.**132** (0.091)	**0**.**126** (0.102)	0.137 (0.060)	0.160 (0.122)
Staphyloma	0.110 (0.078)	**0**.**173** (0.131)	**0**.**193** (0.153)	0.444 (0.133)	0.413 (0.484)

The use of bold numerals serves to indicate which of the two approaches is more accurate

In simulation, SF MAEs range from 147 to 401 $\upmu \hbox {m}$, while their SD varies between 123  $\upmu \hbox {m}$ and 355  $\upmu \hbox {m}$. EF MAEs range from 33 to 38  $\upmu \hbox {m}$ for ellipsoidal shapes, while that of the pointed eye reaches 744  $\upmu \hbox {m}$. In the case of the printed ellipsoidal shapes, the EF MAE ranges from 165 to 197  $\upmu \hbox {m}$, while for non-ellipsoidal shapes, SF MAEs range from 126 to 499  $\upmu \hbox {m}$.

**Fig. 5:**
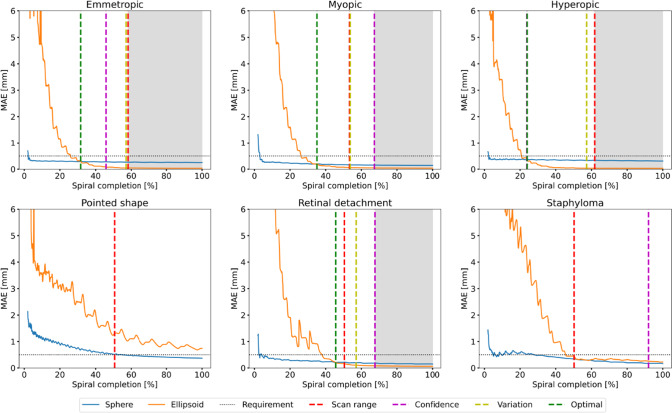
Simulation results of sphere and ellipsoid fitting for all six designed shapes. The points at which the three transition criteria and the optimal ground truth transition (green) are reached are highlighted as vertical dashed lines. The background color serves to indicate the transition from a sphere (white) to an ellipsoid (gray). For the pointed shape and the staphyloma, there is no optimal transition because the sphere fit is always more accurate than the ellipsoid fit. The dashed horizontal line indicates the required model accuracy of 0.5 $\hbox {mm}$

The MAE of the fitted models during the experiments in simulation are displayed in Fig. 5. The results indicate that the model transition criteria accurately determine whether to transition or not, as they only occur when EF produces lower MAEs than SF. However, the transition occurs 21.9 to 37.8% after the optimal transition, contingent upon the fulfillment of all three transition criteria.

**Fig. 6:**
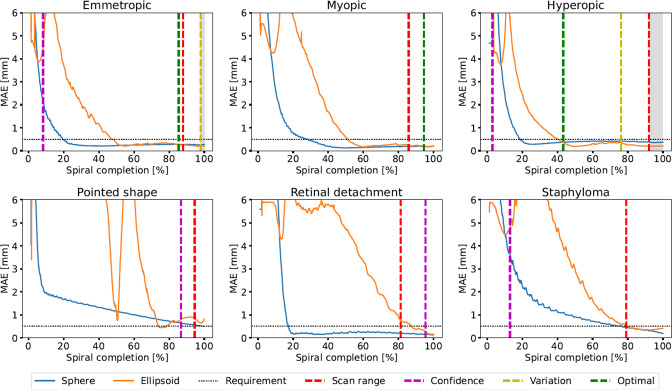
Real-world results of sphere and ellipsoid fitting for all six printed shapes

In the real-world experiment, the criteria demonstrated efficacy in predicting whether to transition or not for all shapes except the myopic eye (Fig. 6), for which the criteria failed to predict the optimal transition occurring at 94.5% of spiral completion. The model transition error is at its highest (48.9%) for the hyperopic eye and at its lowest (12.4%) for the emmetropic eye. In the case of pathological eye shapes, there is no optimal transition as the sphere fit is always more accurate than the ellipsoid fit.

On the three porcine eyes SF FMAEs of 0.290 $\hbox {mm}$, 0.221 $\hbox {mm}$, and 0.330 $\hbox {mm}$ are achieved. The fitted radii are 11.3 $\hbox {mm}$, 12.4 $\hbox {mm}$, and 12.7 $\hbox {mm}$. The EF FMAEs for the porcine eyes are 0.173 $\hbox {mm}$, 0.154 $\hbox {mm}$, and 0.181 $\hbox {mm}$. The retinas of the pigs were observed to be free from any pathological abnormalities. In the absence of the ground truth and the MAE, the evaluation of the model transition was not possible.

## Discussion

An intraoperative retinal model should be able to represent the retina in a way that allows safe surgery without collisions. Based on surgeon interviews and the assessment of intraoperative OCT images, we estimate the minimal distance between the surgical instrument and the retina during vitreous shaving procedures to be approximately 0.5 $\hbox {mm}$. Therefore, we define the acceptable maximum error to be below this threshold. Achieving an MAE of less than 0.5 $\hbox {mm}$ for all six eye shapes indicates that the selected fitting methods are effective in approximating realistic eye shapes.

In general, our global model has demonstrated the capacity to extrapolate beyond the scanned area, encompassing the periphery and the region in close proximity to the trocar, as evidenced by the low MAEs. In the majority of cases where the eye geometry is regular, that is to say, emmetropic, myopic or hyperopic, the results demonstrate that the EF performance on a global scale is superior to that of the SF. However, it is noteworthy that even for ellipsoidal shapes, the SF FMAEs are relatively small (below 100 $\upmu \hbox {m}$), which can be attributed to the fact that, at the local level, an ellipsoid can be approximated by a sphere.

In contrast to the EF FMAEs, the results for SF FMAEs are comparable in both the real world and in simulation, which is consistent with our hypothesis that SF is more robust to noise and real-world data. In the case of the pointed shape, which is composed of a paraboloid and a semi-elllipsoid, the EF is having difficulties and is outperformed by the SF, which achieved an MAE of 499 $\upmu \hbox {m}$. The EF FMAE, however, is relatively small (162 $\upmu \hbox {m}$), indicating that the EF could be fitted locally with a high degree of accuracy. The findings demonstrate that an ellipsoid is inadequate for representing certain pathological shapes, necessitating the development of a more sophisticated model to accurately approximate local features such as detachments, the fovea, or the optic disc.

In the simulation, the average of the MMAEs is 46 $\upmu \hbox {m}$ which is attributable to error sources such as artificial noise and discretization. In the real world, the MMAEs are considerably higher due to a number of factors, including calibration, robot motion, segmentation and synchronization.

The results of this study demonstrate that the model transition criteria provide a reliable indication of the optimal timing for transitioning. However, it should be noted that the applicability of the method is influenced by the selection of hyperparameters. It is beneficial to have all three transition criteria in place, for instance, in the case of the staphyloma, the model transition is only halted by the temporal variation criterion.

The evaluation could be enhanced by the placement of fiducial markers within the shapes, thereby superseding the registration of the shapes with ICP. The results obtained on porcine eyes demonstrate the feasibility of our modeling pipeline when applied to real tissue. Further work will include closed sky experiments and the construction of a reference model for the porcine eyes based on, for example, micro-CT.

A drawback of commonly used EF algorithms is that they minimize the algebraic residual, but not the geometric distance of the measurement points from a best-fitting ellipsoid. The Cayley transform approach converts the EF problem with nonlinear constraints into one with simple linear constraints. Cayley transform ellipsoid fitting (CTEF) is invariant under rotations and translations and is guaranteed to converge [18]. However, these favorable properties come at the cost of worse accuracy and runtime on our data. An advancement to our EF would be the use of extended or unscented Kalman filters [22], which can handle nonlinear constraints while allowing for online model updating. The presented EF method, however, is sufficiently rapid to meet time constraints, with a maximum runtime of less than 0.1 $\hbox {s}$. A key advantage of our method is the availability of a preliminary spherical model at the onset of core vitrectomy. By the time critical steps are undertaken, a more accurate ellipsoid model is established. Once real-time A-scan streaming is available, the model can be employed to define a virtual boundary and other robotic assistance functions for ophthalmic surgery.

## Conclusion

In this work, we have developed a global eye model that is generated intraoperatively from iiOCT data. The proposed model transitions from a sphere to an ellipsoid, maximizing model reliability based on the available data. The model has shown to be able to approximate different retinal geometries corresponding to realistic eye shapes, meeting the accuracy and time requirements for clinical use. In conclusion, our modeling has great potential for improving, assisting and automating PPV and will be incorporated into the GEYEDANCE research platform in further research.

## Data Availability

The representative eye shapes and the eye modeling, including fitting and transition, will be made available to readers upon request.
